# Transcriptomics‐based analysis of the mechanism by which Wang-Bi capsule alleviates joint destruction in rats with collagen‐induced arthritis

**DOI:** 10.1186/s13020-021-00439-w

**Published:** 2021-04-12

**Authors:** Haiyang Shu, Hanxiao Zhao, Yingjie Shi, Cheng Lu, Li Li, Ning Zhao, Aiping Lu, Xiaojuan He

**Affiliations:** 1 The Second Clinical College, Guangzhou University of Chinese Medicine, Guangzhou, 510006 China; 2 Institute of Basic Research in Clinical Medicine, China Academy of Chinese Medical Sciences, Beijing, 100700 China; 3 Shanghai Innovation Center of TCM Health Service, Shanghai University of Traditional Chinese Medicine, Shanghai, China; 4 Law Sau Fai Institute for Advancing Translational Medicine in Bone & Joint Diseases, School of Chinese Medicine, Hong Kong Baptist University, Kowloon Tong, Hong Kong

**Keywords:** Wang-Bi capsule, Rheumatoid arthritis, Joint destruction, Molecular mechanism, Transcriptomic

## Abstract

**Background:**

Rheumatoid arthritis (RA) is a chronic autoimmune disease accompanied with joint destruction that often leads to disability. Wang-Bi capsule (WB), a traditional Chinese medicine-based herbs formula, has exhibited inhibition effect on joint destruction of collagen-induced arthritis (CIA) animal model in our previous study. But its molecular mechanisms are still obscure.

**Methods:**

CIA rats were treated intragastrical with WB for eight weeks, and the effect of joints protection were evaluated by hematoxylin and eosin (H&E) staining, safranin O fast green staining, tartrate-resistant acid phosphatase (TRAP) staining and micro‑CT scanning analysis. The transcriptomic of tarsal joints were used to investigate how WB alleviated joint destruction.

**Results:**

The histological examination of ankle joints showed WB alleviated both cartilage damage and bone destruction of CIA rats. This protective effect on joints were further evidenced by micro-CT analysis. The transcriptomic analysis showed that WB prominently changed 12 KEGG signaling pathways (“calcium signaling pathway”, “cAMP signaling pathway”, “cell adhesion molecules”, “chemokine signaling pathway”, “complement and coagulation cascades”, “MAPK signaling pathway”, “NF-kappa B signaling pathway”, “osteoclast differentiation”, “PI3K-Akt signaling pathway”, “focal adhesion”, “Gap junction” and “Rap1 signaling pathway”) associated with bone or cartilage. Several genes (including Il6, Tnfsf11, Ffar2, Plg, Tnfrsf11b, Fgf4, Fpr1, Siglec1, Vegfd, Cldn1, Cxcl13, Chad, Arrb2, Fgf9, Egfr) regulating bone resorption, bone formation and cartilage development were identified by further analysis. Meanwhile, these differentially expressed genes were validated by real-time quantitative PCR.

**Conclusions:**

Overall, the protective effect of WB treatment on joint were confirmed in CIA rats, and its basic molecular mechanisms may be associated with regulating some genes (including Il6, Tnfsf11, Ffar2 and Plg etc.) involved in bone resorption, bone formation and cartilage development.

**Supplementary Information:**

The online version contains supplementary material available at 10.1186/s13020-021-00439-w.

## Introduction

Rheumatoid arthritis (RA) is a chronic, autoimmune disease that is accompanied by persistent inflammation of synovial tissue as well as joint destruction. Joint destruction often interferes with physical function and even results in disability, which further affects productivity and quality of life. Approximately 80% of patients have deformed joints, and 40% lose work due to disability within 10 years of disease onset [1]. Many earlier studies indicated that joint destruction was correlated with signs and symptoms of inflammation, and some patients with RA have benefited from anti-inflammatory therapies. However, joint destruction still occurs with a high incidence after anti-inflammatory treatment. Accumulating evidence in recent years has revealed that inflammatory activity and joint destruction may be dissociated from one another [2, 3]. Thus, anti-inflammatory drugs are not sufficient to protect joints, and agents that can directly inhibit joint destruction are essential for RA patients. Although several biologic agents have exhibited articular protection to some extent, how to effectively block joint destruction and protect joint function in RA is still a great challenge for clinical doctors because of the lack of sufficient joint protection agents to date [4–6].

Wang-Bi capsule (WB), approved by the China Food and Drug Administration (approval ID of CFDA: Z20080096), is a traditional Chinese medicine-based herbal formula. It has been used for RA treatment in China for many years. WB displayed joint protective effects in a RA mouse model, and it could regulate osteoclast-osteoblast functions and inhibit bone destruction in joints in our previous study [7]. Icariin and paeoniflorin are both active ingredients of WB. Icariin abrogated osteoclast differentiation in vitro and inhibited bone loss by regulating the coupling process of osteogenic and osteoclastic activity in rats with experimental osteoporosis [8–11]. Paeoniflorin suppresses osteoclast differentiation by controlling the receptor activator of nuclear factor-κ B ligand (RANKL)/osteoprotegerin (OPG) ratio [12]. Furthermore, icariin and paeoniflorin were also proven to regulate the activity of chondrocytes and inhibit articular cartilage degradation [13, 14]. These studies hinted that WB was a promising candidate drug for joint protection in RA patients. However, it is still unclear how WB inhibits joint destruction in RA. In this study, we aimed to demonstrate the joint protective effects of WB in a CIA rat model and explore the molecular mechanism of action via transcriptomic analysis.

## Methods

### Animals

Male 6- to 8-week-old Sprague Dawley rats (Vital River Laboratory Animal Technology Co. Ltd., Beijing, China) were used for this study, and all rats were housed in a specific pathogen-free facility with free access to sterilized water and chow. In addition, the experiments were reviewed and approved by the Research Ethics Committee of the Institute of Basic Theory of Chinese Medicine, China Academy of Chinese Medical Sciences.

### Induction of the CIA model

The CIA model was established in line with the method reported in our previous study [15]. The rats were given an intradermal injection of 200 µl of an emulsified mixture of equal proportions bovine collagen type II and incomplete Freund’s adjuvant (Chondrex, Redmond, WA, USA) on day 1. In addition, the rats were immunized on day 7 by another injection of 100 µl of the emulsified mixture.

## Treatment

WB was provided by Liaoning China Resources Benxi Sanyao Co., Ltd. (Liaoning, China, No. 20180205). The rats were randomly divided into three groups after successful induction of the CIA model, with eight rats in each group: the model group, WB group (WB 0.74 g/kg/d, equal to that used in RA patients) and normal group. The rats in the WB group were intragastrically administered WB for 8 weeks after successful induction of the CIA model. The rats in the normal and model groups were administered an equal volume of double-distilled water in the same way. All animals were sacrificed at the end of the experiment. The sera were harvested for enzyme-linked immunosorbent assay (ELISA). The hind limbs were collected for micro-CT analysis, and the ankle joints were removed for histological analysis. The tarsal joints were separated from the hind limbs and used for transcriptomics analysis and real-time quantitative PCR (RT-qPCR).

### ELISA

The serum levels of type I collagen N-terminal propeptide (PINP) and C-terminal telopeptide of type I collagen (CTX-1) were detected using commercially available ELISA kits (Sino-uk bio, Beijing, China). The procedures were performed according to the manufacturer’s instructions.

### Histological analysis

The ankle joints of rats were fixed in 10% phosphate-buffered formalin for 3 days and then decalcified in 10% EDTA (pH = 7.2). Section (4 μm) were stained with hematoxylin and eosin (H&E) for histopathological assessment, and the histopathological characteristics were scored on a scale of 0–4 using the criteria described in a previous study [15]. In addition, sections were stained with safranin O fast green (Solarbio, Beijing, China) for cartilage observation, and cartilage damage was evaluated with the following criteria: 0 = normal, 1 = mild loss of safranin O staining with no obvious chondrocyte loss, 2 = moderate loss of staining with focal mild chondrocyte loss, 3 = marked loss of staining with multifocal marked chondrocyte loss, and 4 = severe diffuse loss of staining with multifocal severe chondrocyte loss [16]. TRAP staining was performed using a TRAP staining kit (Sigma, St. Louis, MO, USA) to visualize osteoclast formation, and the number of osteoclasts per bone surface around the taluses was determined [17].

### Micro‑CT scanning analysis

The hind paws and femurs were scanned for reconstruction into a three-dimensional (3-D) structure with a SKYSCAN 1174 micro-CT (Bruker, Belgium). The hind paws were analyzed to test the bone volume (BV)/tissue volume (TV) ratio, and the femurs were analyzed to determine the bone mineral density (BMD) of the periarticular bone, trabecular number (Tb.N), trabecular pattern factor (Tb.Pf) and trabecular separation (Tb.S) [18].

### Transcriptome of the tarsal joints

Three tarsal joints were sampled per group (normal group, model group and WB group) at the end of the experiment. The skin, muscle, tendon and ligament were removed, but bone tissue was included in each sample [19]. The samples were stored at − 80 °C. Total RNA was extracted from the tissues using TRIzol (Invitrogen, Carlsbad, CA, USA) according to the instructions in the product manual. Subsequently, total RNA was qualified and quantified using a NanoDrop (NanoDrop, Madison, USA) and an Agilent 2100 bioanalyzer (Agilent, Santa Clara, USA). The products were enriched with PCR to create the final cDNA library for mRNA sequencing. The constructed library was amplified to make DNA nanoballs (DNBs) with more than 200 copies of DNA per molecule. The DNBs were loaded into the patterned nanoarray, and single-end 100-base reads were generated by sequence-based synthesis. The qualified libraries were subjected to paired-end sequencing on the BGISEQ-500 System (BGI-Shenzhen, China).

The sequencing data were filtered with SOAPnuke (v1.5.2), and clean reads were obtained and stored in FASTQ format. The clean reads were mapped to the reference genome using HISAT2 (v2.0.4). Bowtie2 (v2.2.5) was applied to align the clean reads to the reference coding gene set, and the expression levels of genes were calculated with RSEM (v1.2.12).

Differential expression analysis was performed using DESeq2 (v1.4.5) with a fold change > 2.0 and a P-value < 0.01. To gain insight into the change in phenotype, KEGG (https://www.kegg.jp/) enrichment analysis of annotated differentially expressed genes was performed with Phyper (https://en.wikipedia.org/wiki/Hypergeometric_distribution) based on a hypergeometric test. The significance levels of pathways were filtered by P-value, with a rigorous threshold (P-value < 0.01) in the Bonferroni test. Network analysis was performed by ingenuity pathway analysis (IPA, Ingenuity Systems).

### RT-qPCR

RNA (2 µg per reaction) was reverse transcribed to cDNA using TRUEscript RT MasterMix (Aibosen Biotechnologies Co., Ltd., Beijing, China). qPCR was performed with 2× SYBR Green qPCR Master Mix (Biomake, Houston, TX, USA). PCR was initiated at 95 °C for 5 min, and the cycling conditions were 95 °C for 10 s, 60 °C for 15 s and 75 °C 15 s (40 cycles). Samples were analyzed in triplicate and normalized by subtraction of the sample mean Ct value from the mean Ct value of the housekeeping gene GAPDH. The primer information is shown in Additional file 1: Table S1.

### Statistical analysis

GraphPad Prism v.6 (GraphPad Software, San Diego, CA, USA) was used for statistical analyses. Ranked data including histological scoring were assessed with the Kruskal-Wallis test followed by a multiple comparisons test. Measurement data including RT-qPCR were analyzed by ANOVA, followed by Bonferroni’s multiple comparison test. P < 0.05 was identified significant.

## Results

### WB inhibited joint destruction in rats with CIA

**Fig. 1:**
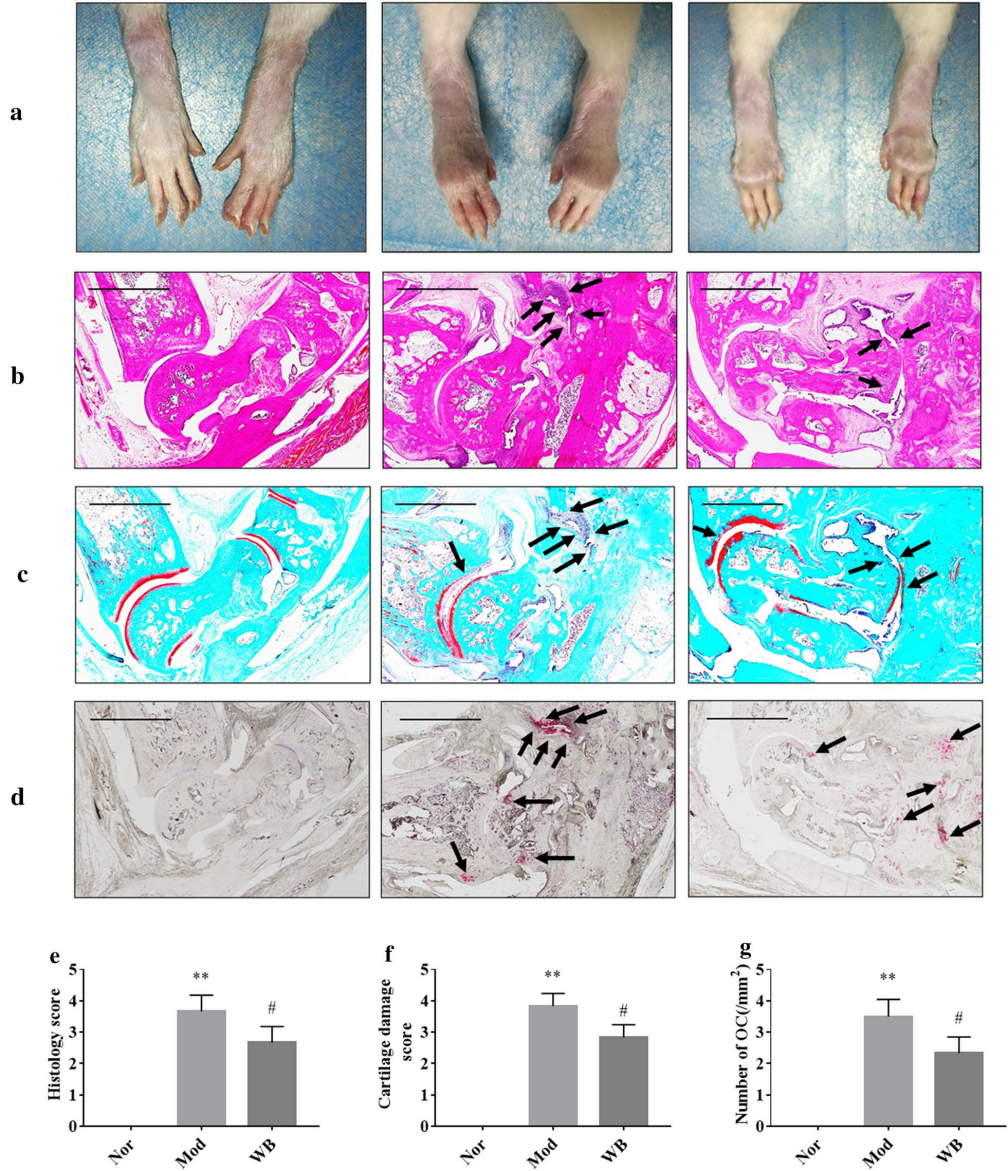
WB inhibited joint destruction in rats with CIA. **a** Representative photographs of ankle joints from each group. Representative images of H&E staining (**b**), safranin O fast green staining (**c**) and TRAP staining (**d**). The black arrows mark bone destruction, cartilage destruction or osteoclast-positive staining. Original magnification ×6.6, the scale bar was 2 mm. Semiquantitative analysis of histology scores by H&E staining (**e**), cartilage damage by safranin O fast green staining (**f**) and the number of osteoclasts by TRAP staining (**g**). Values are the mean ± SD, n = 6. **P < 0.01 compared with the Nor group. ^#^P < 0.05 compared with the Mod group. Abbreviation: *Nor* normal, *Mod* model

The joints of rats in the model group displayed severe swelling and deformation, and WB treatment remarkably improved these symptoms compared with those of model group rats (Fig. 1a). To further evaluate the effect of WB on joint destruction in rats with CIA, H&E staining was performed. The results showed no detectable pathological changes in the rats in the normal group. In contrast, the rats in the model group exhibited severe inflammatory cell infiltration and bone and cartilage damage, whereas these pathological changes in the WB group were significantly relieved compared with those in the model group (Fig. 1b, e). Next, safranin O fast green staining was used to further investigate the changes in articular cartilage. The results showed severe cartilage damage in the model group compared with the normal group, represented by a markedly decreased amount of cartilage matrix. However, the cartilage damage in the WB group exhibited obvious mitigation compared with that of the model group (Fig. 1c, f). Finally, TRAP staining was performed to detect the number of osteoclasts. We did not detect positive staining for osteoclasts in the normal group, but a large number of osteoclasts were observed in the model group. In contrast, the number of osteoclasts with positive staining in the WB group was significantly decreased compared with that in the model group (Fig. 1d, g). These results indicated that WB could effectively relieve bone and cartilage destruction in rats with CIA.

### WB increased the PINP/CTX-1 ratio of rats with CIA

**Fig. 2:**
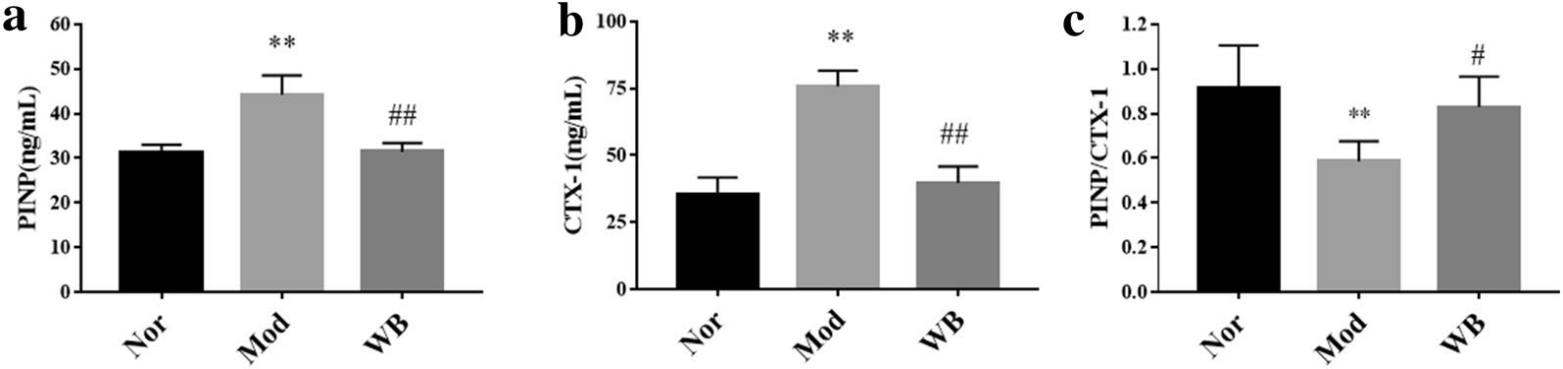
The effect of WB on serum levels of PINP and CTX-1 in rats with CIA. **a** The serum level of the bone formation marker PINP in different groups. **b** The serum level of the bone resorption marker CTX-1 in different groups. **c** The PINP/CTX-1 ratio. Values are the mean ± SD, n = 6. **P < 0.01 compared with the Nor group. ^#^P < 0.05, ^##^P < 0.01 compared with the Mod group. Abbreviation: *PINP *type I collagen N-terminal propeptide, *CTX-1* C-terminal telopeptide of type I collagen

PINP and CTX-1 are important biochemical markers of bone formation and bone resorption, respectively. The serum levels of PINP and CTX-1 were all significantly increased in the model group compared with the normal group (Fig. 2a, b). However, the PINP/CTX-1 ratio was higher in the WB group than in the model group (Fig. 2c), which provided evidence that WB could promote bone formation in rats with CIA.

### WB inhibited bone erosion and bone loss in rats with CIA

**Fig. 3:**
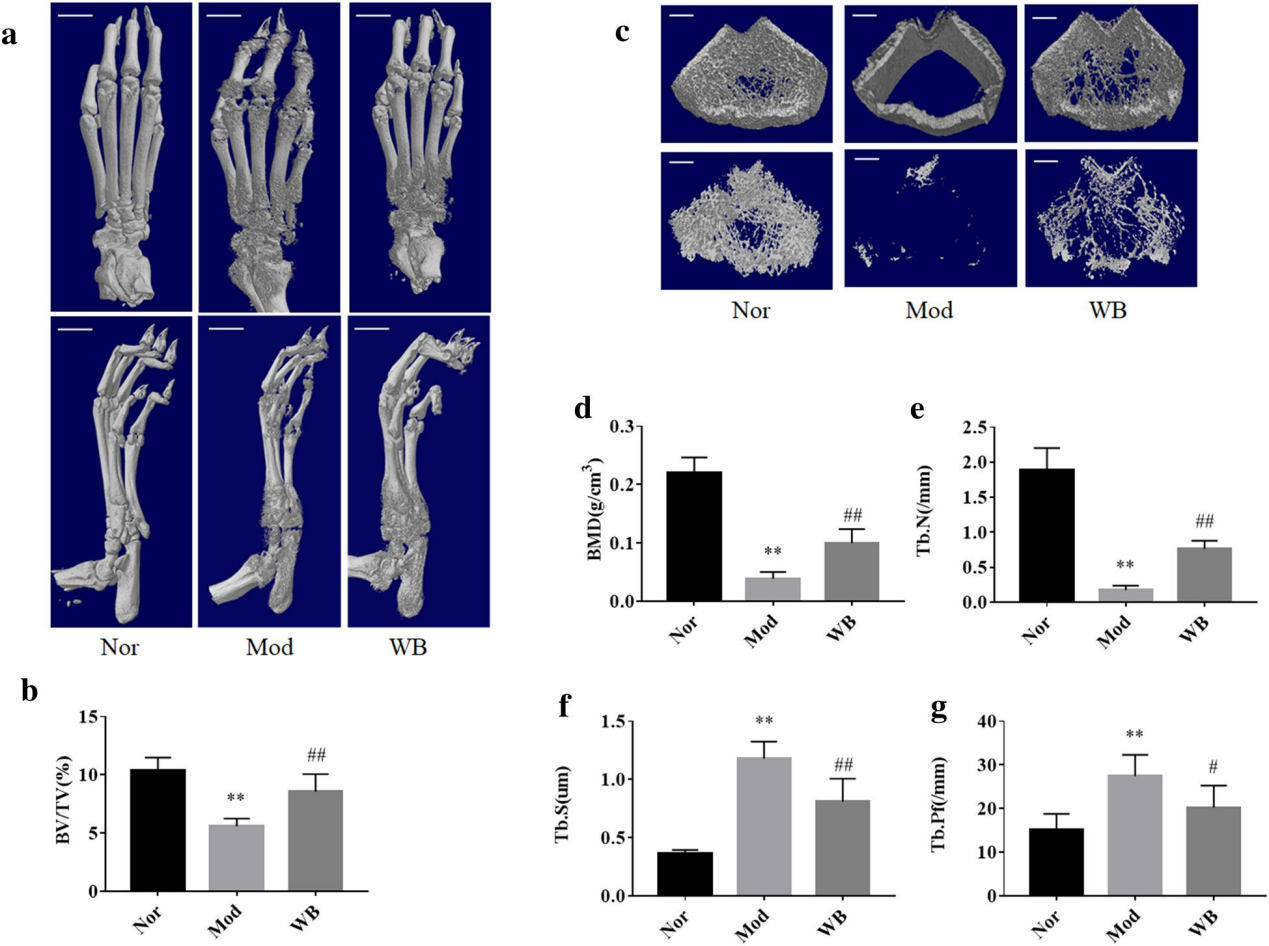
WB improved the bone microstructure of rats with CIA. **a** Representative bone micro-CT images of the hind paws. The scale bar was 5 mm. **b** BV/TV of the hind paws. **c** Representative bone micro-CT images of the distal femur. The scale bar was 1 mm. **d** BMD, **e** Tb.N, **f** Tb.S, and **g** Tb.Pf. Values are the mean ± SD, n = 6. **P < 0.01 compared with the Nor group. ^#^P < 0.05, compared with the Mod group. Abbreviation: *BV/TV* bone volume/tissue volume, *BMD *bone mineral density, *Tb.N *trabecular number, *Tb.S *trabecular separation, *Tb.Pf *trabecular pattern factor

Micro-CT and 3D image analysis were used to examine the effect of WB on bone erosion and bone microstructure. The CT analysis showed that bone erosion was substantially more severe in the model group than in the normal group. After WB treatment, bone erosion was inhibited (Fig. 3a). Furthermore, the BV/TV ratio in the WB group was significantly increased compared with that in the model group (Fig. 3b). In addition, the distal femur microstructure was reconstituted (Fig. 3c). We found that BMD and Tb.N in the model group were obviously lower than those in the normal group, whereas these two parameters in the WB group were significantly increased compared with those in the model group (Fig. 3d, e). Both the Tb.S and Tb.Pf of the model group increased compared to those of the normal group, and WB treatment significantly decreased the Tb.S and Tb.Pf (Fig. 3f, g). Overall, these results indicated that WB could inhibit bone erosion and bone loss in rats with CIA.

## WB regulated signaling pathways associated with bone or cartilage

**Fig. 4:**
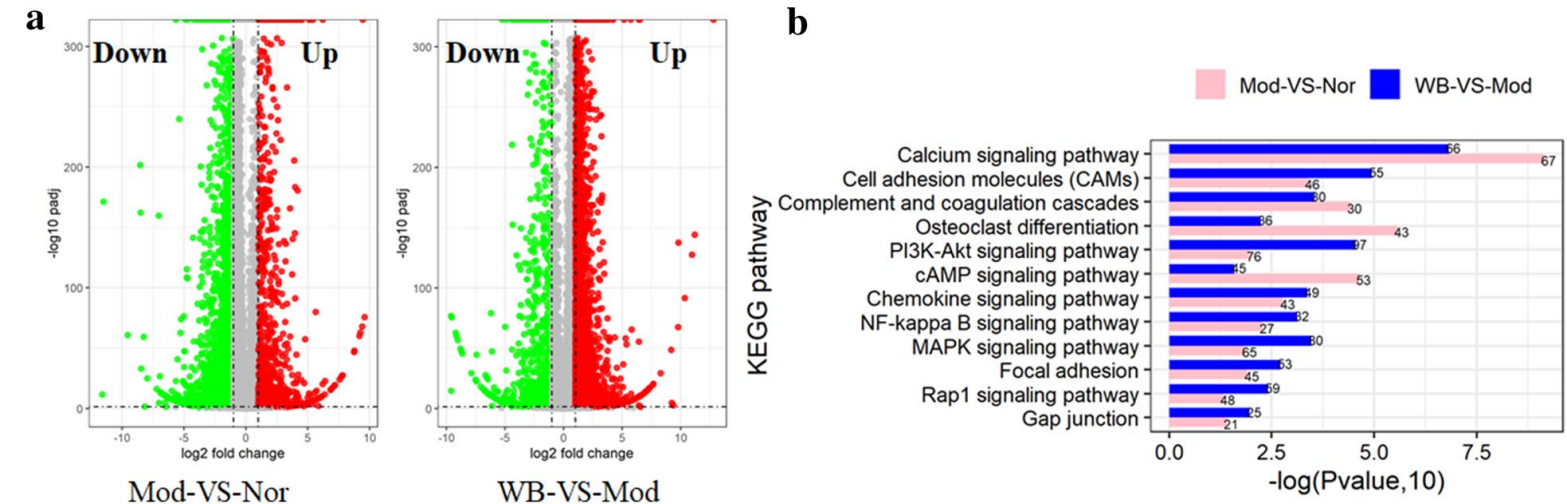
Analysis of DEGs. **a** Volcano plot of DEGs. A total of 2988 DEGs were downregulated and 802 DEGs were upregulated in Mod-VS-Nor, while 3362 DEGs were upregulated and 777 DEGs were downregulated in WB-VS-Mod. **b** Twelve signaling pathways associated with bone or cartilage were significantly enriched by KEGG analysis. Abbreviation: DEGs: differentially expressed genes

Although WB exhibited a protective effect on joints, the potential mechanism was still unclear. Therefore, we wanted to reveal the mechanism via transcriptomic analysis. A total of 3790 differentially expressed genes (DEGs) were identified in Mod-VS-Nor; 2988 DEGs showed downregulated expression, and 802 DEGs showed upregulated expression. A total of 4139 DEGs were identified in WB-VS-Mod; 3362 DEGs showed upregulated expression, and 777 DEGs showed downregulated expression (Fig. 4a). By KEGG analysis of DEGs in Mod-VS-Nor and WB-VS-Mod, we found 12 obviously enriched (P value < 0.05) signaling pathways associated with bone or cartilage, including “calcium signaling pathway”, “cell adhesion molecules (CAMs)”, “complement and coagulation cascades”, “osteoclast differentiation”, “PI3K-Akt signaling pathway”, “cAMP signaling pathway”, “chemokine signaling pathway”, “NF-kappa B signaling pathway”, “MAPK signaling pathway”, “focal adhesion”, “Rap1 signaling pathway” and “gap junction” (Fig. 4b).

### WB regulated the levels of some genes that controlled bone and cartilage function in rats with CIA

**Fig. 5:**
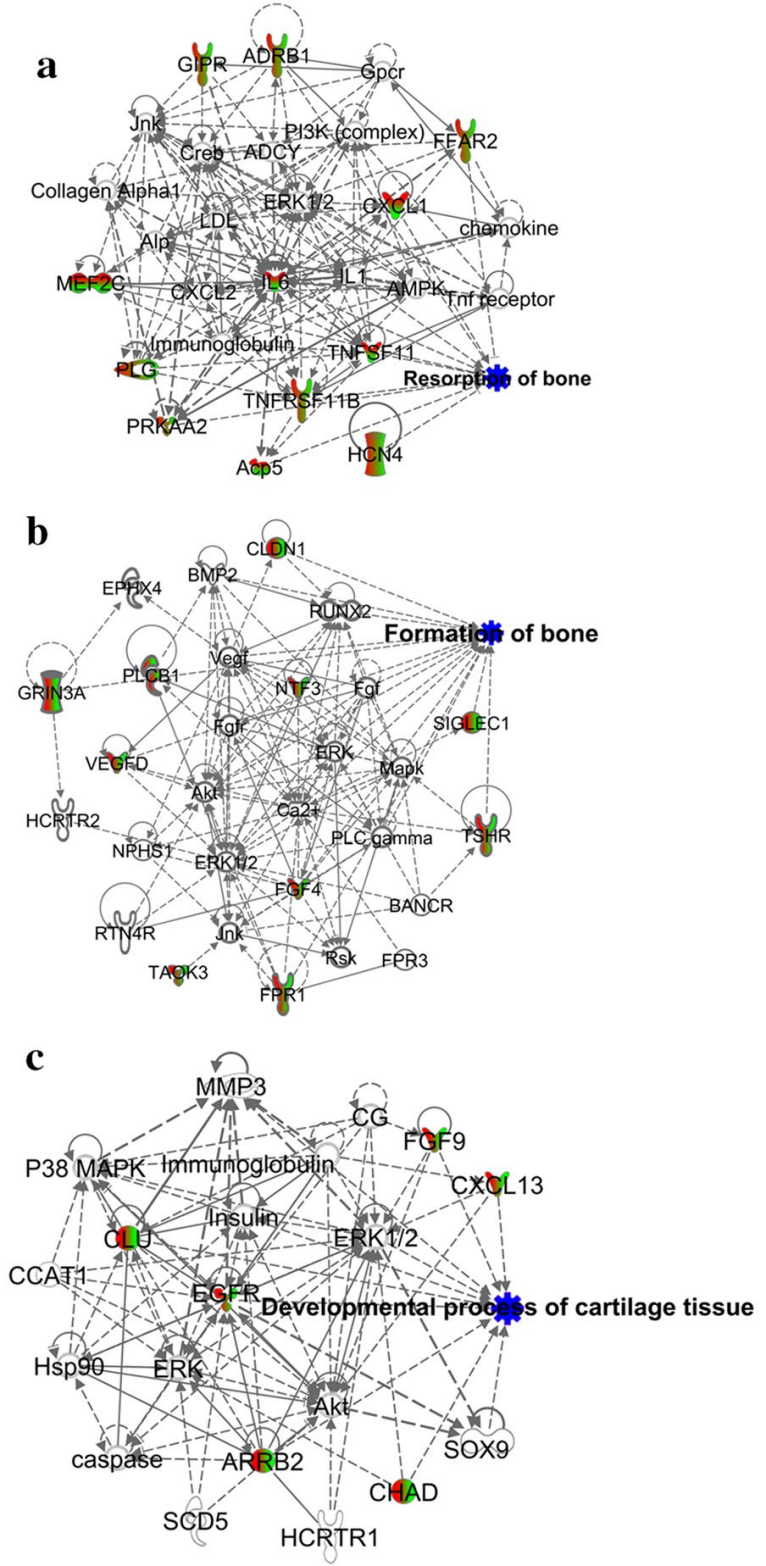
The functional networks revealed by gene analysis. The network of genes associated with bone resorption (**a**), bone formation (**b**) and cartilage development (**c**). The red color on the top of the molecule indicates that the transcript level of the gene is upregulated in Mod-VS-Nor, and the green color on the bottom of the molecule indicates that the transcript level of the gene is downregulated in WB-VS-Mod. In addition, the red color on the left indicates that the transcript level of gene is upregulated in WB-VS-Mod, and the green color on the right indicates that the transcript level of gene is downregulated in Mod-VS-Nor. The blue color represents the biofunction

Having identified the 12 signaling pathways that regulated bone and cartilage function, we next focused on the DEGs belonging to these signaling pathways. A total of 373 DEGs from Mod-VS-Nor and 425 DEGs from WB-VS-Mod belonged to the 12 pathways associated with bone or cartilage identified by KEGG analysis, and there were 238 overlapping DEGs between these two groups. These genes were input into IPA, and three functional networks related to bone or cartilage were generated: the bone resorption network, bone formation network and cartilage development network (Fig. 5). Moreover, the levels of genes promoting bone resorption (including Il6, Tnfsf11, Cxcl1, Acp5 and Mef2c) were elevated in the model group but were suppressed by WB treatment, while the levels of genes inhibiting bone resorption (including Ffar2, Plg, Tnfrsf11b, Hcn4, Adrb1, Prkaa2 and Gipr) were decreased in the model group but were increased by WB treatment (Additional file 1: Table S2). The transcript levels of genes promoting bone formation (including Fgf4, Fpr1, Siglec1, Vegfd, Cldn1, Taok3, Grin3a, Tshr, Plcb1 and Ntf3) were decreased in the model group but were increased by WB treatment (Additional file 1: Table S3). Genes involved with cartilage development (including Cxcl13, Chad, Arrb2, Fgf9, Egfr and Clu) exhibited low levels in the model group but higher levels after WB treatment (Additional file 1: Table S4).

### DEG validation by RT-qPCR

**Fig. 6:**
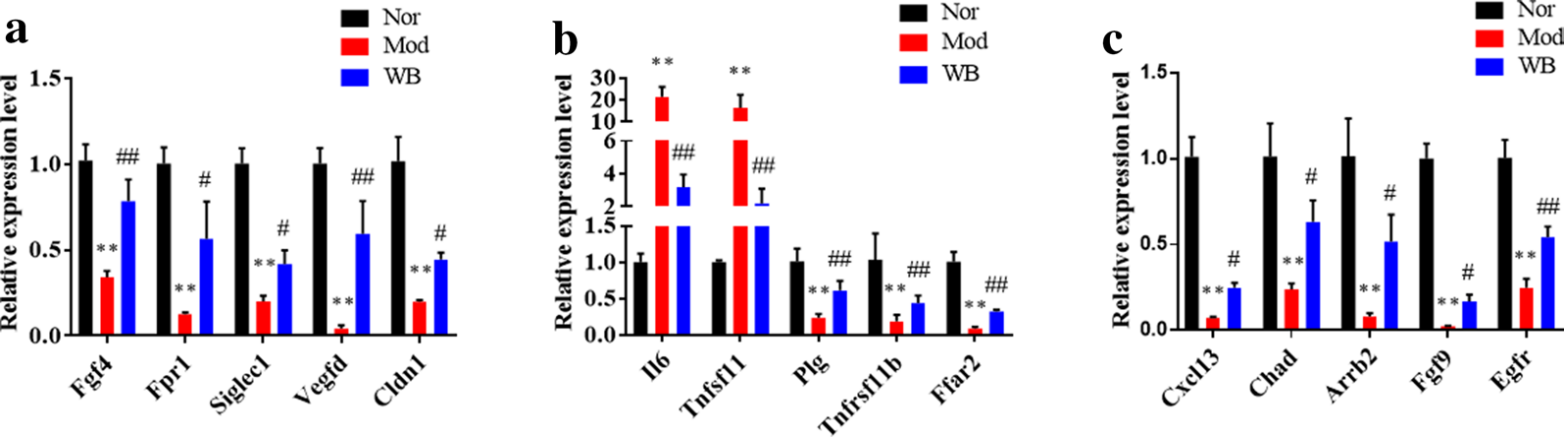
DEG validation by RT-qPCR. **a** The mRNA levels of DEGs related to bone formation. **b** The mRNA levels of DEGs related to bone resorption. **c** The mRNA levels of DEGs related to cartilage development. Values are the mean ± SD, n = 3. **P < 0.01 compared with the Nor group. ^#^P < 0.05, compared with the Mod group

The top 5 DEGs (according to fold change) associated with bone resorption, bone formation and cartilage development were chosen for validation by RT-qPCR. The mRNA expression levels of genes promoting bone formation (Fgf4, Fpr1, Siglec1, Vegfd, and Cldn1) were remarkably decreased in the model group compared with the normal group, but they were all significantly increased in the WB group compared with the model group (Fig. 6a). The levels of Il6 and Tnfsf11 were much higher in the model group than in the normal group; however, they were significantly downregulated by WB treatment. In contrast, the levels of genes suppressing osteoclast differentiation, such as Ffar2, Plg, and Tnfrsf11b, were markedly elevated by WB treatment (Fig. 6b). Furthermore, the transcript levels of genes controlling cartilage development, such as Cxcl13, Chad, Arrb2, Fgf9 and Egfr, were decreased in the model group, whereas they were markedly upregulated in the WB group (Fig. 6c). The changes in the transcript levels of these genes were consistent with the tendencies observed in the transcriptome analysis.

## Discussion

Joint destruction is one of the predominant types of damage underlying RA disease. Accumulating evidence has proven a positive correlation between joint destruction and disability, therefore, it is essential to consider whether therapeutic agents have a direct impact on progressive joint damage. Our previous studies revealed that WB not only inhibited the inflammatory response but also alleviated joint destruction [7]. In this study, we mainly focused on WB’s protective effects on joints. WB treatment obviously improved the histopathological manifestations of CIA in rats, including inhibition of bone destruction and cartilage damage. Bone loss often occurs in RA, which increases fracture risk. Rats with CIA displayed high bone turnover and osteoporosis in previous studies [20]. In our study, the rats with CIA also exhibited high bone turnover and presented elevated serum levels of both PINP (a bone formation marker) and CTX-1 (a bone resorption marker). WB treatment increased the PINP/CTX-1 ratio, which indicated that WB could inhibit bone loss in rats with CIA. Furthermore, the protective effect of WB treatment on bone was also evidenced by the micro-CT examination results.

The mechanisms by which WB protects joints are complicated because of the intricate ingredients of WB. Accordingly, transcriptomic analysis of the tarsal joints was performed to evaluate the impacts of WB on cartilage and bone. The KEGG analysis of DEGs identified several signaling pathways associated with bone and cartilage. We know that the balance of osteoclasts and osteoblasts is important for bone function, and “osteoclast differentiation” is a core factor that controls osteoclast formation [21]. The KEGG database shows that pathways including the “calcium signaling pathway”, “MAPK signaling pathway”, “PI3K-Akt signaling pathway” and “NF-kappa B signaling pathway” participate in the induction of “osteoclast differentiation”. Some studies have also revealed that these pathways are deeply involved in regulating osteoclast differentiation or activation, which further impacts bone resorption [22–25]. “Focal adhesion” and the “MAPK signaling pathway” are important parts of the “Wnt signaling pathway”, which plays an essential role in osteoblast differentiation. Furthermore, osteochondrogenitor cells express both Runx2 and Sox9, and “cAMP signaling” modifies these transcription factors and drives differentiation to osteoblasts or chondrocytes [26, 27]. In addition, cAMP induces RAP1 activation, which promotes integrin clustering and cell adhesion to the bone matrix [28, 29]. The migration and maturation of osteoclasts and osteoblasts are important processes in bone remodeling. Pathways including “cell adhesion molecules”, “chemokine signaling pathway” and “gap junctions” all help to facilitate these processes [30–34]. “Complement and coagulation cascades” are seldom reported in the context of regulating bone remodeling in RA. However, several studies have hinted at a role for these pathways. For example, FVIIIKO and PAR1KO mice displayed a lower bone/tissue volume ratio and a smaller number of bone trabeculae than wild-type mice [35, 36]. Overall, we found that WB could regulate these signaling pathways, which helped us understand how WB influenced the joints of rats with CIA.

To further dissect the mechanism by which WB protects bone and cartilage, we analyzed these DEGs (belonging to the signaling pathways mentioned above) from several profiles. Several DEGs (Il6, Tnfsf11, Cxcl1, Acp5, and Mef2c) promoting bone resorption were first identified. Their expression levels were all decreased by WB treatment. IL-6 is a proinflammatory cytokine, and multiple studies in recent years have shown that it is also an important factor for promoting osteoclast differentiation. The inhibition of IL-6 retarded the progression of joint damage in RA independent of its anti-inflammatory effects, which suggested disassociation of the link between inflammation and joint destruction [37, 38]. RANKL (encoded by Tnfsf11) is absolutely necessary for osteoclast differentiation, and both Cxcl1 and Mef2c can enhance RANKL-induced osteoclast differentiation [39–41]. Furthermore, Acp5, encoding TRAP (a marker of osteoclasts), was also suppressed by WB treatment. In addition, the mRNA levels of genes (Ffar2, Plg, Tnfrsf11b, Hcn4, Adrb1, Prkaa2 and Gipr) that suppressed bone resorption were elevated by WB treatment. Plasminogen (Plg) is an important regulator of bone metabolism that has not been described in RA. Exogenous Plg suppressed RAW264.7 cell differentiation to osteoclasts. Moreover, Plg-/- mice displayed a low BMD [42]. OPG (encoded by Tnfrsf11b), a soluble decoy receptor for RANKL, can inhibit bone loss by controlling osteoclast differentiation. AMPKα2 (encoded by Prkaa2) could inhibit RANKL signaling [43]. Ffar2 and Hcn4 are both seldom reported in RA studies, but gene knockdown experiments have shown decreased formation of osteoblasts or BMD [44, 45]. Stimulation of the sympathetic nervous system by beta-2 adrenergic receptor (β2AR) plays an important role in mediating bone remodeling [46]. β1AR (encoded by Adrb1) has also been proven to mitigate negative changes in cancellous bone microarchitecture and increase the bone mass of mice [47]. Glucose-dependent insulinotropic peptide (GIP), an intestinally secreted hormone, could decrease bone resorption. Excess signaling during GIPR (encoded by Gipr) activation resulted in increased bone mass [48]. In this study, we found that rats with CIA displayed low transcript levels of Ffar2, Plg, Tnfrsf11b, Hcn4, Adrb1, Prkaa2 and Gipr compared with those of normal rats, whereas the levels of all these genes transcripts were significantly increased by WB treatment, which demonstrated that WB treatment could effectively inhibit bone resorption in rats with CIA.

In addition to influencing genes regulating bone resorption, WB also regulated several genes regulating bone formation. Bone morphogenetic protein family members (BMPs) are important signaling molecules regulating bone formation. Fgf4, with a very high transcript level after WB treatment, could cooperate with BMP-2-induced bone formation, and administration of FGF4 could increase bone mass in rats [49, 50]. Similar to FGF4, PLCβ1 can also cooperate with BMPs. Increased expression of PLCβ1 induced by BMP-2 could promote osteoblast differentiation *in vitro* [51]. Activation of the N-formyl peptide receptor (FPR) can trigger multiple biochemical cascades and eventually lead to cellular activation. Its role during the differentiation of osteocytes has received attention in recent years. Activation of FPR1 could promote osteogenesis and mineralization, but activation of FPR2 or FPR3 could not [52]. The anabolic process is an important process of bone remodeling. For example, CD169 (encoded by Siglec1) could provide pro-anabolic support during the process of bone repair [53]. VEGF-D could promote the formation of mineralized nodules and accelerate bone formation, which provides another option to promote bone formation [54]. Claudins (Cldns) are major components of tight junctions. In addition to functioning as tight junctions, they have been found to play roles in cell proliferation and differentiation. Cldn1 was downregulated during osteoclast differentiation but upregulated during osteoblast differentiation, and Cldn1 knockdown decreased the expression of Runx2 [55]. Proteins encoded by genes such as Taok3, Tshr, Grin3a and Ntf3 could also promote bone formation by enhancing osteoblast differentiation or mineralization [56–60]. The transcript levels of these genes were elevated by WB treatment, which might contribute to bone formation through multiple pathways.

Articular cartilage damage is a very important pathological change during joint destruction in RA. Cartilage tissue is composed of extracellular matrix (ECM) that is synthesized by chondrocytes. The degradation of cartilage ECM also provides an opportunity for the hyperplastic synovium to directly invade subchondral bone. Although cartilage tissue has limited self-repair ability, recent studies have shown that cartilage contains a population of stem cells or progenitor cells that facilitate cartilage development and repair [27]. Thus, cartilage may benefit from WB treatment through several genes, such as Cxcl13, Chad, Arrb2, Fgf9, Egfr and Clu, that regulate cartilage development. Cartilage homeostasis is disrupted when damage occurs. Chemokines and chondroadherin (CHAD) could contribute to the maintenance of cartilage homeostasis by promoting ECM production or assembly [57, 61]. FGF9 is a member of the fibroblast growth factor (FGF) family, and studies have shown that it could enhance chondrogenesis in dental pulp stem cells [62]. However, the role of FGF9 with respect to the articular cartilage damage underlying RA has not been well described. The epidermal growth factor receptor (EGFR) system plays important roles in multiple processes, such as the differentiation and proliferation of osteoclasts, osteoblasts and chondrocytes [63]. Both beta-arrestin-2 (ARRB2) and clusterin (CLU) could inhibit the inflammatory response of chondrocytes, but CLU could also influence the proliferation and differentiation of chondrocytes [64, 65]. In this study, we found that the expression of Cxcl13, Chad, Arrb2, Fgf9, Egfr and Clu was obviously suppressed in rats with CIA, but WB treatment increased it to a certain degree.

## Conclusions

Overall, the protective effect of WB on joints was confirmed in rats with CIA, and its basic molecular mechanisms may be associated with regulation of the expression levels of some genes (including Il6, Tnfsf11, Ffar2, Plg, Tnfrsf11b, Fgf4, Fpr1, Siglec1, Vegfd, Cldn1, Cxcl13, Chad, Arrb2, Fgf9, and Egfr) involved in bone resorption, bone formation and cartilage development.

## Supplementary Information

**Additional file 1: Table S1.** Primers used for RT-qPCR. **Table S2.** Fold change of DEGs related to bone resorption regulated by WB. **Table S3.** Fold change of DEGs related to bone formation regulated by WB. **Table S4.** Fold change of DEGs related to cartilage development regulated by WB.

## Data Availability

The datasets used and analyzed during the current study are available from the corresponding author on reasonable request.

## Abbreviations

RA: Rheumatoid arthritis
WB: Wang-Bi capsule
CIA: Collagen-induced arthritis
RANKL: Receptor activator of nuclear factor-κ B ligand
OPG: Osteoprotegerin
PINP: Type I collagen N-terminal propeptide
CTX-1: C-terminal telopeptide of type I collagen
BV/TV: Bone volume/tissue volume
BMD: Bone mineral density
Tb.N: Trabecular number
Tb.Pf: Trabecular pattern factor
Tb.S: Trabecular separation
DEGs: Differentially expressed genes
