# Supplementary material for: Transcriptomics‐based analysis of the mechanism by which Wang-Bi capsule alleviates joint destruction in rats with collagen‐induced arthritis
Source: Chin Med. 2021 Apr 12;16:31. doi: 10.1186/s13020-021-00439-w (PMC8042720; doi:10.1186/s13020-021-00439-w)
Supplement: Supplementary file 1 — Additional file 1: Table S1. Primers used for RT-qPCR. Table S2. Fold change of DEGs related to bone resorption regulated by WB. Table S3. Fold change of DEGs related to bone formation regulated by WB. Table S4. Fold change of DEGs related to cartilage development regulated by WB. [file 13020_2021_439_MOESM1_ESM.docx]

**Additional file**

**Additional file 1: Table S1. Primers used for RT-qPCR.**

| Siglec1 | FORWARD | CGGCGTGGCATCCTCTTGTG |
| --- | --- | --- |
|  | REVERSE | GAGTTGGAGGCTCTGCTGACTTTC |
| Cldn1 | FORWARD | CTTCTGGGTTTCATCCTGGCTTCG |
|  | REVERSE | CCTGAGCAGTCACGATGTTGTCC |
| Fgf4 | FORWARD | TGTGCCTTTCTTTACCGACGAGTG |
|  | REVERSE | CTTCTTGGTCCGCCCGTTCTTAC |
| Plg | FORWARD | AGCACACGAAGAACGAATCCTTGG |
|  | REVERSE | TTAGCTTCAGCAGGGCAATGTCAG |
| Fpr1 | FORWARD | GGTTCATCCTTGGGTTCAGCACTC |
|  | REVERSE | GGGACGGCTGGATTTGATAAGACC |
| Vegfd | FORWARD | CTCTGTCCTGTTGACATGCTGTGG |
|  | REVERSE | GAGAGCGGGTTCCTGGAGGTAAG |
| IL-6 | FORWARD | ACTTCCAGCCAGTTGCCTTCTTG |
|  | REVERSE | TGGTCTGTTGTGGGTGGTATCCTC |
| Tnfsf11 | FORWARD | GAGCGAAGACACAGAAGCACTACC |
|  | REVERSE | GAGCCACGAACCTTCCATCATAGC |
| Tnfrsf11b | FORWARD | TGTGTCCCTTGCCCTGACTACTC |
|  | REVERSE | CTCGGTTGTGGGTGCGGTTG |
| Ffar2 | FORWARD | TGCACCATCGTCATCATCGTTCAG |
|  | REVERSE | ACCAGGCACAGCTCCAGTCG |
| Cxcl13 | FORWARD | ACACCTGCACCCTTTCTTAATCCC |
|  | REVERSE | TCGAGTGTCTCCTCTCAGCTACTG |
| Chad | FORWARD | CCTGAGCAAACTTCGGGTGGTG |
|  | REVERSE | CAGCCAGAGGGTCTCCAGGTATC |
| Arrb2 | FORWARD | GGAACTCCGTGCGGCTTATCATC |
|  | REVERSE | GAGGAAGTGGCGTGTGGTTTCAG |
| Fgf9 | FORWARD | CCAGGAAAGACCACAGCCGATTC |
|  | REVERSE | GTCCACTGTCCACACCACGAATG |
| Egfr | FORWARD | GACTGTGTCTCCTGCCAGAATGTG |
|  | REVERSE | TCCACAAACTCCCTCGGTTCCC |
| GAPDH | FORWARD | GACATGCCGCCTGGAGAAAC |
|  | REVERSE | AGCCCAGGATGCCCTTTAGT |

**Additional file 1: Table S2. DEGs related to bone resorption regulated by WB.**

| NO. | Gene Symbol | Fold change (P-value<0.01) | |
| --- | --- | --- | --- |
|  |  | Mod-VS-Nor | WB-VS-Mod |
| 1 | Il6 | 49.87 | -74.03 |
| 2 | Tnfsf11 | 6.23 | -3.86 |
| 3 | Ffar2 | -6.15 | 4.41 |
| 4 | Plg | -5.70 | 8.51 |
| 5 | Tnfrsf11b | -5.06 | 3.12 |
| 6 | Hcn4 | -4.99 | 10.63 |
| 7 | Cxcl1 | 4.17 | -4.63 |
| 8 | Acp5 | 3.25 | -2.38 |
| 9 | Adrb1 | -2.91 | 3.78 |
| 10 | Prkaa2 | -2.77 | 2.48 |
| 11 | Gipr | -2.46 | 4.47 |
| 12 | Mef2c | 2.36 | -2.35 |

**Additional file 1: Table S3. DEGs related to bone formation regulated by WB.**

| NO. | Gene Symbol | Fold change (P-value<0.01) | |
| --- | --- | --- | --- |
|  |  | Mod-VS-Nor | WB-VS-Mod |
| 1 | Fgf4 | -20.97 | 17.88 |
| 2 | Fpr1 | -5.31 | 2.17 |
| 3 | Siglec1 | -4.63 | 4.86 |
| 4 | Vegfd | -3.92 | 3.56 |
| 5 | Cldn1 | -2.53 | 5.74 |
| 6 | Taok3 | -2.43 | 2.83 |
| 7 | Grin3a | -2.41 | 3.78 |
| 8 | Tshr | -2.36 | 4.32 |
| 9 | Plcb1 | -2.13 | 2.28 |
| 10 | Ntf3 | -2.13 | 2.69 |

**Additional file 1: Table S4. DEGs related to cartilage development regulated by WB.**

| NO. | Gene Symbol | Fold change (P-value<0.01) | |
| --- | --- | --- | --- |
|  |  | Mod-VS-Nor | WB-VS-Mod |
| 1 | Cxcl13 | -19.16 | 2.10 |
| 2 | Chad | -12.21 | 7.11 |
| 3 | Arrb2 | -5.17 | 3.07 |
| 4 | Fgf9 | -4.69 | 3.39 |
| 5 | Egfr | -4.00 | 2.14 |
| 6 | Clu | -2.89 | 2.53 |
